# Editorial: Emerging talents in frontiers in pharmacology: pharmacogenetics and pharmacogenomics 2022

**DOI:** 10.3389/fphar.2023.1307602

**Published:** 2023-10-27

**Authors:** Sylvia D. Klomp, Ana Alfirevic

**Affiliations:** ^1^ Leiden University Medical Center, Clinical Pharmacy and Toxicology, Leiden, Netherlands; ^2^ Department Pharmacology and Therapeutics, University of Liverpool, Liverpool, United Kingdom

**Keywords:** pharmacogenetics, pharmacogenomics, early career researcher, publications, future

Pharmacogenetics/phrmacogenomics (Pgx) is a mature study and in several healthcare systems, including the US, Europe, and Asia, Pgx has moved from discovery research to implementation into clinical practice. The Pgx research spans across almost all clinical specialties, ranging from infection to transplantation and oncology to neurology. The Frontiers current themed Research Topic was dedicated to highlighting the emerging early career researcher (ECR) talents including many PhD students, who are undertaking research in the field of Pgx. The papers in this Research Topic showcase the quality and diversity of ECR research across the field of Pgx. The articles included range from basic research to implementation of Pgx into clinical practice, and they reflect several Research Topic of current interest in the field including cancer, drug safety with examples from HLA immune mediated adverse drug reactions and organ directed toxicity exemplified by statin-induced myotoxicity in patients with cardiovascular diseases.

Starting with the research by Habil et al., who investigated the novel variant NAT1*14B important in the metabolism of N-acetylation of β-naphthylamine in Chinese hamster ovary cells. They demonstrated lower rates of metabolism and affinity to acetyl coenzyme A for NAT1*14B compared with the known NAT1*4 variant.

In study by Hurkmans et al., the authors utilised retrospective osteosarcoma cohorts from the Netherlands, Spain, the United Kingdom and Australia to identify genetic factors associated with early disease progression. They investigated the SLC7A8 locus and found LAT2-mediated uptake of doxorubicin in functional studies.

In a recruit-by-genotype trial, the effect of the LILRB5 T/T genotype was studied to examine its role in immune response Tornio et al. It was found that during atorvastatin treatment, the LILRB5 genotype was associated with an increase in creatine kinase and a decrease in total and non-HDL cholesterol.

Dai et al. reviewed the influence of miRNAs on ferroptosis in tumours. They demonstrated that there was a large opportunity for the use of miRNAs in cancer treatment, because different miRNAs were found to be involved in the regulation of tumour occurrence and development via the ferroptosis pathway.

Two articles in this themed Research Topic focused on the use of pharmacogenetic methods in the clinic. In a review by Manson et al., genotyping of HLA risk alleles was compared with patch testing as a diagnostic tool for cutaneous hypersensitivity reactions caused by anti-seizure medications. It was found that there were limited data available, but the authors confirmed yet again that clinicians could use HLA-B genotyping for SJS/TEN for several drugs in specific populations, but interestingly, patch testing was more reliable for DRESS caused by carbamazepine and phenytoin.

The last article in this Research Topic studied different *-allele tools for long-read sequencing data of CYP2C19 genotyping and phenotype prediction Graansma et al. They found that it was possible to correctly predict the CYP2C19 phenotypes using three different tools but concluded that in clinical practice the added value of this approach is limited.

**FIGURE 1:**
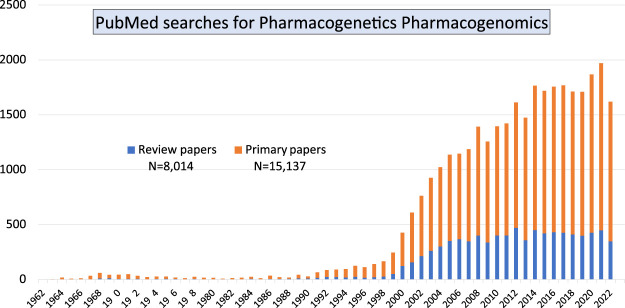
Pubmed searches using two terms pharmacogenetics and pharmacogenomics indicate that primary research in the field is still on the increase compared with review articles/secondary publications.

Together, those papers show the diversity of both, basic research which focused on the advancement of knowledge, and of applied research directed towards finding a solution to clinical problems. Our PubMed searches using the terms “pharmacogenetics pharmacogenomics” showed that over the past 60 years there is still a continuously increasing number of primary research papers being published in the field of Pgx as well as a steady number of review articles (Figure 1). The large increase in publications starting from the year 2000 coincided with the completion of the human genome sequencing. But the number of papers is not decreasing, year after year there are more than 1,500 papers published. Involvement of early career researchers working in the field is important as it indicates continuous funding streams for the Pgx research.

Interestingly, in this Research Topic there are no papers studying education in Pgx. This was surprising, because Pgx education is particularly important to facilitate implementation of Pgx into clinical practice. This was already recognised in 2017 when pharmacists were surveyed for their perception of Pgx (Bank et al., 2017). It was found that over the previous 6 months only ∼15% ordered a PGx test, mostly because they felt that they lacked knowledge to do so. The results of the PREPARE study (Swen et al., 2023) showed that pharmacists were improving their competence, but apart from pharmacists and pharmacologists, all healthcare professionals would need to be able to interpret pharmacogenetic results. In 2014 in the United Kingdom, Genomics Education Programme (GEP) was developed in collaboration with National Health Service (NHS) England and Genomics England. To date, the GEP has funded more than 1,500 NHS professionals to participate in the Masters in Genomic Medicine programme designed to provide healthcare professionals with a multidisciplinary perspective on genomics and its applications in healthcare (https://www.genomicseducation.hee.nhs.uk/about-us/masters-in-genomic-medicine/).

In this Research Topic, ECR demonstrated a bright future ahead in the Pgx research. In diverse patient populations and multiple clinical specialties Pgx testing can be implemented to personalise treatment. Ever since pharmacology has been studied, it has been known that drug response is complex. Genetics could contribute to a large proportion (up to 91%) of drug response, but at present in clinical practice only 20%–40% of response can be explained (Matthaei et al., 2015; Lauschke and Ingelman-Sundberg, 2019). There are many other factors that influence efficacy and toxicity of drug response. Some of the hurdles for implementation of pharmacogenetics in clinics already highlighted in literature are missing heritability (Klein and Zanger, 2013; van der Lee et al., 2021), phenoconversion (Shah and Smith, 2015; Klomp et al., 2020) and epigenetics (Manikandan and Nagini, 2018; Zanger et al., 2018). Finally, it is clear that we have to move from single variant genotyping to panel testing and sequencing of multiple variants. Furthermore, using clinical and environmental data incorporated into electronic medical records accompanied by the advanced computing capability and big data integration using machine learning and artificial intelligence, will open new avenues of research in Pgx for patient benefit.
